# Identity development, attraction, and behaviour of heterosexually identified men who have sex with men: scoping review protocol

**DOI:** 10.1186/s13643-023-02355-6

**Published:** 2023-09-30

**Authors:** Andrew D. Eaton, Travis R. Scheadler, Cara Bradley, Lauren B. McInroy, Oliver W. J. Beer, Erin Beckwell, Adam Busch, Paul A. Shuper

**Affiliations:** 1 Faculty of Social Work – Saskatoon Campus, University of Regina, Saskatoon, SK Canada; 2 Factor-Inwentash Faculty of Social Work, University of Toronto, Toronto, ON Canada; 3 College of Social Work, The Ohio State University, Columbus, OH USA; 4 Dr. John Archer Library, University of Regina, Regina, SK Canada; 5 School of Health Professionals, The University of Plymouth, Plymouth, UK; 6 HQ Toronto, Toronto, ON Canada; 7 Centre for Addiction and Mental Health, Institute for Mental Health Policy Research, Toronto, ON Canada; 8 Dalla Lana School of Public Health, University of Toronto, Toronto, ON Canada

**Keywords:** Heterosexually identified men who have sex with men, Sexual identity-behaviour discordance, Relationships, Social work, Psychology

## Abstract

**Background:**

Heterosexually identified men who have sex with men (H-MSM) are distinct from other heterosexual men and from gay, bisexual, and other sexual minority men. Specifically, H-MSM experience discordance between their sexual identity (i.e., heterosexual) and behaviours (i.e., sexual encounters with other men). This sexual identity-behaviour discordance can create barriers to obtaining healthcare and social support. Understanding and accepting H-MSM as they self-identify may be necessary to implement effective public health and psychosocial interventions. The aim of the present study is to provide an overview of research on H-MSM.

**Methods:**

A scoping review will be conducted to identify and describe the identity development, attraction, and behaviour of H-MSM. This scoping review will also identify and describe current trends related to the recruitment of H-MSM and recommend directions for future research. Searches will be conducted in Academic Search Complete, APA PsychInfo, CINAHL Plus with full text, Education Research Complete, Gender Studies Database, GenderWatch, Health Source: Nursing/Academic Edition, LGBTQ + Source, MEDLINE, Psychology and Behavioral Sciences Collection, SocINDEX with full text, Sociological Collection, Social Work Abstracts, ProQuest Dissertations and Theses, and ResearchGate. Primary research studies published in peer-reviewed journals will be included. Dissertations and theses that include primary research on H-MSM will also be included. Reference lists, experts in the field, preprint servers, and relevant conferences will also be consulted for extant and in-progress literature. Two reviewers will independently pilot the data extraction form and conduct the title and abstract screening, with consultation from a research librarian. Seven reviewers will then conduct the full-text article screening. Thematic content analysis will guide the review; through independent review and reviewer meetings, themes and subthemes will be identified and reported from the extracted literature.

**Discussion:**

This is the first known knowledge synthesis on H-MSM, seeking to better understand sexual identity-behaviour discordance amongst cisgender men. We anticipate that a theoretical framework of H-MSM’s sexuality, internal processes, and behaviours will be constructed from this review. Alongside implications for further research with H-MSM, this review may be relevant to sexually transmitted infection public health and to clinicians working in the field of male sexuality.

**Systematic review registration:**

Open Science Framework: https://doi.org/10.17605/OSF.IO/MVY9H

**Supplementary Information:**

The online version contains supplementary material available at 10.1186/s13643-023-02355-6.

## Background

Estimates suggest that heterosexually identified men who have sex with men (H-MSM) account for approximately 8% of sexually active cisgender males in North America [1, 2]. This population experiences discordance between their sexual identity and behaviour. That is, despite identifying as heterosexual, these cisgender men engage in behaviours associated with non-heterosexuality. Specifically, they engage in sexual behaviours with other men, actions associated with non-heterosexual identities (e.g., gay, bisexual, pansexual).

There is some contention around the identity of H-MSM as, unlike their sexual minority counterparts, they experience incongruity between their sexual identity and behaviours. Whilst some scholars have provided evidence that H-MSM are not closeted gay or bisexual men [2–7], other scholars have argued that adverse childhood experiences related to homophobia, religious upbringings, conservatism, and conversion therapy, among others, may explain why some H-MSM are reluctant to identify as non-heterosexual [2, 8–11]. Accepting these individuals’ identity as heterosexual and acknowledging heterosexuality can fluctuate are important to effectively engaging with this population and promoting healthy behaviours [12].

Research has documented disparities between H-MSM and other heterosexual men. For example, previous research on H-MSM suggests some H-MSM view relationships with other men as strictly sexual and view their relationships with women as primary and intimate [9, 6, 13]. Evidence indicates that H-MSM engage in high rates of behaviours that can present a high risk of HIV/AIDS and other sexually transmitted infections [14, 15]. Mostly-heterosexual-identified men also report greater perceived stress than heterosexual men who only have other-sex partners [16]. This study also found that H-MSM’s perceived stress-mediated increases in symptoms of depression [16]. H-MSM also have worsened levels of physical health, depression, anxiety, and suicidal ideation compared to heterosexual men who only have other-sex partners [17]. In a nationally representative sample of 11,522 cisgender young adults, Caplan found that straight men with discordant sexual behaviors (i.e., actions that do not align with their sexual orientation) experience more depressive symptoms than straight men with concordant sexual behaviors (i.e., actions that do align with their sexual orientation) [18]. Sexual orientation discordance has also been associated with increases in depressive symptomology among mostly-heterosexual male adolescents with same-sex partners [19]. Similarly, adolescents who identify as heterosexual with same-sex partners are more likely to have a suicide plan than those who exclusively have other-sex partners [20]. H-MSM may also be more likely than those with concordant sexual orientations and behaviors to use alcohol and inhalants [21]. Moreover, H-MSM tend to perceive themselves as less happy than the average person [22] and report lower levels of social support than heterosexual men [23].

In comparison to gay and bisexual men, H-MSM may also have greater levels of guilt and shame as well as poorer coping abilities and sexual communication in their relationships [2, 6, 9, 13, 24–27]. Research in Puerto Rico shows that H-MSM have poorer knowledge of sexual health than gay and bisexual men [28]. Parent and colleagues found that MSM who present as heterosexual were less likely than other MSM to get tested for HIV [29]. The authors purported that heterosexually presenting and identified MSM may be concerned that HIV testing will ‘out’ them [29]. However, HIV testing in all MSM is imperative for decreasing rates of HIV transmission and providing appropriate care for those who have a positive HIV serostatus.

Given these disparities, there is a need for researchers and practitioners to develop a deeper understanding of the experiences of H-MSM. A literature synthesis has not yet been conducted on this population. A scoping review may be the most appropriate method of synthesis on research related to H-MSM given the lack of existing knowledge in this area [30]. Therefore, this scoping review will collate and assess the extant literature related to the population. More specifically, the aim of this scoping review is to identify the concepts, methods, findings, and gaps in peer-reviewed research related to H-MSM. This includes research on the identity development, attraction, and behaviour (e.g., sexual engagements, modes of communication) of H-MSM as each of these concepts can aid in the understanding of H-MSM and help researchers, healthcare practitioners, and community organisers to design and implement interventions to promote healthy lifestyle behaviours and greater overall well-being.

## Methods

A scoping review is most suitable for the present study as the research topic is broad, includes more than one research question, and explores an area that includes relatively little research [30]. As such, this protocol followed guidelines established by the preferred reporting item for systematic and meta-analyse extension for a protocol (PRISMA-P; supplementary file 1). However, given that the current study is a scoping review protocol and not a systematic review or meta-analysis, the PRISMA extension for scoping reviews (PRISMA-ScR) will be followed for reporting the results. In addition, this proposed scoping review will follow Arksey and O’Malley’s framework, which includes identifying the research question, identifying relevant studies, selecting eligible studies, charting data, and collating, summarising, and reporting the results [31].

### Identifying the research question

The objective of this scoping review is to identify and describe current knowledge about the constructs of identity development, attraction, and behaviour of heterosexually identified men who have sex with men (H-MSM), alongside recruitment considerations and directions for future research. Therefore, the review has the following research questions:

1. What is known about the identity development, attraction, and behaviour of H-MSM?
  a How have H-MSM developed their sexual identity?
  b Who are H-MSM attracted to, and how do they define that attraction?
  c What behaviours do H-MSM engage in regarding sexual engagements?
2. How have H-MSM been recruited in previous primary research studies? 

#### Eligibility of the research question

This review determined the eligibility of its research questions based on the PCC (Population, Concept, Context) elements. Our scoping review will cover studies that include H-MSM in their sample (Population). More specifically, H-MSM are adults (aged 18 and over) who self-identify as straight and/or heterosexual yet have engaged in sex with at least one other man during their lifetime. Importantly, studies may include other participants in their sample in addition to H-MSM as long as H-MSM are included in the sample and not combined with any other group. That is, studies will not be included if analyses combined H-MSM with another group (e.g., bisexual MSM) as this would prevent a study from drawing conclusions specific to H-MSM.

In addition, our scoping review will include studies that examine identity development, attraction, and**/**or behaviour (Concept). Identity development, attraction, and behaviour are three different dimensions of sexuality. Data related to identity development will include how H-MSM make sense of and maintain their heterosexual identity whilst data related to attraction will include what kind of romantic or sexual emotions, feelings, thoughts, and urges H-MSM have toward others. Then, data related to behaviour will include what kinds of actions H-MSM engage in regarding their health, whether by themselves or with others. Insight into each of these dimensions can help us develop a stronger understanding of the experiences of H-MSM. Finally, our study will consider evidence from all countries and settings (Context).

### Identifying relevant studies

Several databases will be used to search for primary, peer-reviewed published research. Specifically, the following databases will be searched for relevant research published from January 1, 2000, onwards: Academic Search Complete, APA PsychInfo, CINAHL Plus with Full Text, Education Research Complete, Gender Studies Database, GenderWatch, Health Source: Nursing/Academic Edition, LGBTQ + Source, MEDLINE, Psychology and Behavioral Sciences Collection, SocINDEX with Full Text, Sociological Collection, Social Work Abstracts, and ProQuest Dissertations and Theses. In addition, relevant research will be identified by screening the reference lists of included studies, contacting experts in the field, searching the abstract books of relevant conferences (e.g., Society for Social Work and Research), scanning the Canadian Institute of Health Research (CIHR) and National Institute of Health (NIH) funding decision databases, reviewing preprint servers (e.g., SocArXiv, ResearchGate), and liaising with the research team’s contacts and other relevant researchers. A university librarian will be consulted throughout the study to assist with identifying relevant literature.

Key terms during the search will include ‘heterosexual men who have sex with men’, ‘heterosexually identified men who have sex with men’, ‘straight men who have sex with men’, sexual identity-behaviour discordance’, ‘dude sex’, ‘bud sex’, ‘heteroflexible men’, ‘sex between straight men’, behaviourally bisexual’, ‘non-gay’, ‘down low’, and ‘heterosexual self-presentation’. These reflect the variety of ways researchers have labeled the identities and behaviours of H-MSM. The search strategy will include these terms as keywords, titles, and subject headings. Additionally, the terms will be combined using Boolean terms (e.g., and, or) and the syntax will be adapted for each database. Texts available in English will be included. No study design exclusions will be applied. Therefore, research can include any study design, including both cross-sectional and longitudinal reports as well as quantitative, qualitative, and mixed methods reports. In addition, only studies published since 2000 will be included to help ensure that these studies are contemporary and best represent the current experiences of H-MSM. Supplementary file 2 contains the draft syntax for one of the databases as an example, alongside the number of articles retrieved from that search.

### Study selection

After retrieving the articles from each of the databases, two reviewers will independently perform the title and abstract screening using Covidence software. These two screeners will be independently performing the screen and will not have access to each other’s results. They will then meet to discuss their reviews and will consult a third reviewer to resolve disagreements as necessary. If an article passes the title and abstract screening but the full text cannot be accessed via the reviewers’ university’s library systems, the original author(s) will be contacted via email to retrieve it. Afterwards, seven reviewers—including the two previous reviewers—will perform full-text screening [32]. The full-text screen will involve assigning full texts so that each full text is read by a minimum of three reviewers. Each article will be assigned a unique number, and a random number table will be utilised to ensure that the articles are randomly distributed and that each full text is assigned to a minimum of three reviewers. The full-text reviewers will work independently without having access to the other reviewers’ results. Each reviewer will refer to the inclusion criteria described below.

#### Inclusion criteria

Studies will be included if they meet the following inclusion criteria:

- Primary research reports that include cisgender H-MSM in the sample. In particular, studies must specifically include cisgender MSM who identify as heterosexual or straight and not just the broader and more general MSM population.
- H-MSM in the sample have a history that includes at least one sexual encounter with another man.
- Study results are available in English.
- Studies published from 2000 to present will be included.

Included articles will be saved in Covidence. Deleted files will be saved in a separate file. The PRISMA flow diagram will be used to present the results from the screening process.

### Charting the data

A standardised data extraction sheet will be used. Extracted data will include author name(s), publication year, origin (if applicable), funding (if applicable), aims/purpose, study population and sample size, methods (including recruitment strategy), details of the intervention (if applicable), outcomes or constructs and how they were assessed, and proposed limitations and future directions. This data extraction form will be piloted by the two reviewers who will conduct the title and abstract screening. After achieving consensus on the title and abstract screen, the two reviewers will each extract data from five articles–randomly selected—to pilot test whether the form’s categories encapsulate article material necessary for this review. Piloting this form will ensure consistency and accuracy between reviewers. The data extraction form may be adjusted based on the feedback from the reviewers. The data extraction form will continuously be revised, as necessary, based on the extracted data. Two reviewers will independently extract all studies included in the final review—without access to each other’s results—and will resolve discrepancies via discussion with a third reviewer involved if necessary.

### Collating, summarising, and reporting of the results

Thematic content analysis will be used to identify themes and subthemes relevant to the research questions [33–35]. More specifically, reviewers trained to conduct thematic content analysis will read through and become familiar with the extracted data. The reviewers will then assign descriptive codes to the data. Next, the reviewers will use focused coding to group similar codes together. Afterwards, the reviewers will examine the codes to identify patterns, which will allow themes and subthemes to emerge from the data. Then, the reviewers will cross-check the themes and subthemes with the data to ensure that the themes and subthemes are appropriate. The themes and subthemes will then be identified and described. A narrative account will then be used to summarise and report the themes and subthemes. All authors will also regularly engage in reflexive meetings to discuss and minimise biases, resolve disagreements, and reach consensus. Quantitative data from studies will be analyzed using numerical counts and tables [36]. Findings will then be used to summarise current literature on H-MSM, identify research gaps and future directions, and reflect on the feasibility of a systematic review or future primary studies. Where appropriate, these findings will be presented in tables and/or graphs.

### Quality assessment

The Mixed Methods Appraisal Tool (MMAT) Version 2018 [37] will be used to assess the quality of evidence of each included study. Two reviewers will independently assign ratings ranging from ≤ 50% (low-quality articles), 51–75% (average quality), and 76–100% (high-quality articles). In alignment with other scoping review protocols [38, 39] and as the MMAT creators discourage article exclusion based on quality [37], articles will be included regardless of score. Proportions of articles in each percentile will be reported, with limitations acknowledged regarding any inclusion of low-quality articles. The two reviewers will not have access to each other’s results. Discrepancies between ratings will be resolved via discussion, involving a third reviewer if necessary.

### Discrepancies between protocol and final scoping review

The final scoping review will note any discrepancies between this protocol and the actual review. The final scoping review will also include reasons and impacts for these discrepancies.

## Discussion

This scoping review aims to map current knowledge on identity development, attraction, and behaviour of heterosexually identified men who have sex with men (H-MSM) alongside methodological (e.g., recruitment) considerations for primary studies with this population. The review team is comprised of social workers, psychologists, and public health professionals who have an interest in developing behavioural and health interventions with H-MSM. The review will endeavour to construct a thematic framework that encompasses the complexity of sexual identity-behaviour discordance amongst cisgender men. This complexity will include the range of service needs that H-MSM may identify, the challenges of defining this population, and differential levels of engagement based on healthcare infrastructure and on country or regions’ policies and services for sexual minority men. The review may provide recommendations for further primary studies with H-MSM as well as preliminary directions for potential interventions that would be of relevance to sexuality researchers and clinicians.

### Supplementary Information

**Additional file 1: Supplementary File 1.** PRISMA-P 2015 Checklist.

**Additional file 2:** **Supplementary File 2.** Search Syntax.

## Data Availability

The dataset that will be used during the current study will be available from the corresponding author on reasonable request.

## Abbreviations

H-MSM: Heterosexually identified men who have sex with men
PRISMA: Preferred Reporting Item for Systematic and Meta-Analyses
PRISMA-P: Preferred Reporting Item for Systematic and Meta-Analyses Extension for a Protocol
PRISMA-ScR: Preferred Reporting Item for Systematic and Meta-Analyses Extension for a Scoping Review
