# Supplementary material for: Identity development, attraction, and behaviour of heterosexually identified men who have sex with men: scoping review protocol
Source: Syst Rev. 2023 Sep 30;12:184. doi: 10.1186/s13643-023-02355-6 (PMC10542689; doi:10.1186/s13643-023-02355-6)
Supplement: Supplementary file 2 — Additional file 2: Supplementary File 2. Search Syntax. [file 13643_2023_2355_MOESM2_ESM.docx]

**Supplementary File 2: Search Syntax**

APA PsychInfo (EBSCOHost)

Date Range: January 1, 2000 – June 24, 2022

Search conducted on June 24, 2022

| Search | Query | Records Retrieved |
| --- | --- | --- |
| #1 | heterosexual men who have sex with men [All Fields] OR heterosexually-identified men who have sex with men [All Fields] OR straight men who have sex with men [All Fields] OR straight-identified men who have sex with men | 1686 |
| #2 | “heterosexual men who have sex with men” [Title] OR “heterosexual men who have sex with men” [SU Subjects] OR “heterosexual men who have sex with men” [DE Subjects] OR “heterosexual men who have sex with men” [Keywords] OR “heterosexual men who have sex with men” [Population] OR “heterosexually-identified men who have sex with men” [Title] OR “heterosexually-identified men who have sex with men” [SU Subjects] OR “heterosexually-identified men who have sex with men” [DE Subjects] OR “heterosexually-identified men who have sex with men” [Keywords] OR “heterosexually-identified men who have sex with men” [Population] OR “straight men who have sex with men” [Title] OR “straight men who have sex with men” [SU Subjects] OR “straight men who have sex with men” [DE Subjects] OR “straight men who have sex with men” [Keywords] OR “straight men who have sex with men” [Population] OR “straight-identified men who have sex with men” [Title] OR “straight-identified men who have sex with men” [SU Subjects] OR “straight-identified men who have sex with men” [DE Subjects] OR “straight-identified men who have sex with men” [Keywords] OR “straight-identified men who have sex with men” [Population] OR “heteroflexible men” OR “heteroflexibilit*” [Title] OR “heteroflexible men” OR “heteroflexibilit*” [SU Subjects] OR “heteroflexible men” OR “heteroflexibilit*” [DE Subjects] OR “heteroflexible men” OR “heteroflexibilit*” [Keywords] OR “heteroflexible men” OR “heteroflexibilit*” [Population] | 16 |
| #3 | “sexual identity-behaviour discordance” OR “sexual identity-behavior discordance” [Title] OR “sexual identity-behaviour discordance” OR “sexual identity-behavior discordance” [SU Subjects] OR “sexual identity-behaviour discordance” OR “sexual identity-behavior discordance” [DE Subjects] OR “sexual identity-behaviour discordance” OR “sexual identity-behavior discordance” [Keywords] OR “behaviourally bisexual” OR “behaviorally bisexual” [Title] OR “behaviourally bisexual” OR “behaviorally bisexual” [SU Subjects] OR “behaviourally bisexual” OR “behaviorally bisexual” [DE Subjects] OR “behaviourally bisexual” OR “behaviorally bisexual” [Keywords] OR “bud sex” OR “bud-sex” [Title] OR “bud sex” OR “bud-sex” [SU Subjects] OR “bud sex” OR “bud-sex” [DE Subjects] OR “bud sex” OR “bud-sex” [Keywords] OR “dude-sex” OR “dude sex” [Title] OR “dude-sex” OR “dude sex” [SU Subjects] OR “dude-sex” OR “dude sex” [DE Subjects] OR “dude-sex” OR “dude sex” [Keywords] OR “down low” OR “down-low” [Title] OR “down low” OR “down-low” [SU Subjects] OR “down low” OR “down-low” [DE Subjects] OR “down low” OR “down-low” [Keywords] OR “non-gay” [Title] OR “non-gay” [SU Subjects] OR “non-gay” [DE Subjects] OR “non-gay” [Keywords] | 97 |
| #4 | S2 and S3 | 111 |
| Subtotal | | 1910 |
| Duplicates | | 149 |
| Total | | 1761 |
| Language: English | | |
